# First prospective, single-arm, multicenter study to evaluate safety and efficacy of the overall thrombectomy system -iNedit, iNdeep, and iNtercept- for acute ischemic stroke. Rationale beyond the study

**DOI:** 10.3389/fneur.2025.1537008

**Published:** 2025-03-05

**Authors:** Luís San Román, Laura Ludovica Gramegna, Sara Pich, Laura Domingo-Rodriguez, Marta Duran, Lluís Duocastella, Juan Macho

**Affiliations:** ^1^Department of Neuroradiology, Hospital Clinic, Barcelona, Spain; ^2^Vall d'Hebron Institut de Recerca, Vall d'Hebron Barcelona Hospital Campus, Barcelona, Spain; ^3^iVascular, Barcelona, Spain

**Keywords:** mechanical thrombectomy, acute ischemic stroke, balloon guide catheter, distal aspiration catheter, endovascular stroke treatment

## Abstract

**Rationale:**

The clinical impact of a novel mechanical thrombectomy strategy, which integrates distal access with flow reversal and flow arrest via a distal balloon, all within a single procedure [Safety and Efficacy of the overall throMbectomy system for sTroke (SEMTiC) strategy], has not been tested.

**Aim:**

The SEMTiC-01 study is the first prospective, multicenter *in vivo* study evaluating the safety and efficacy of the combined thrombectomy system—iNedit, iNdeep, and iNtercept—in patients with acute ischemic stroke.

**Sample size estimates:**

The study was designed with a sequential structure based on the efficacy endpoint (eTICI ≥2b) reported in the literature [71.1% with a 95% confidence interval of (68.5%, 73.8%)]. An interim analysis was set for 115 patients and a final analysis for 225 patients, ensuring 98% power at a one-sided 0.025 significance level, with a 2.6% non-inferiority margin and a 15% assumed withdrawal rate.

**Design:**

SEMTiC-01 is a prospective, multicenter, single-arm, open-label clinical safety and efficacy investigation.

**Outcome:**

Primary efficacy endpoint: expanded treatment in cerebral infarction score (eTICI) ≥2b revascularization within ≤ 3 stent retriever passes. Primary safety endpoint: monitoring serious adverse events within 24 h post-intervention and all-cause mortality at 90 days.

## Introduction

Mechanical thrombectomy (MT) for large vessel occlusion (LVO) strokes has been the subject of multiple randomized clinical trials, which have invariably demonstrated its overwhelming efficacy (1).

The initial key trials (2–5) focused on MT within 6 h of onset. However, the DEFUSE 3 (6) and DAWN (7) studies extended the therapeutic window to 24 h for selected patients with late-onset or wake-up strokes. Their findings were included in the 2018 American Heart Association (AHA)/American Stroke Association (ASA) stroke guidelines (8). MT can be performed with or without prior rt-PA (2).

Currently, significant research efforts are focused on improving the techniques available for performing MT.

Two main techniques for MT are stent retrievers and direct aspiration catheters, often used in combination. Stent retrievers, the most common (3), can be paired with a balloon guide catheter (BGC) to arrest and reverse blood flow during thrombus removal (9). However, the BGC is usually only advanced to the extracranial carotid artery for balloon inflation. There are no currently available mechanical devices that feature a balloon catheter near the distal tip, which can be inflated in the intracranial portion of the vessel. This could potentially increase the system's stability as the balloon would be surrounded by the petrous segment of the carotid encased in bone.

Direct aspiration catheters, while comparable to stent retrievers in terms of achieved revascularization (10), are less commonly used. They can be operated manually or with an aspiration pump via distal access catheters (DACs), with flexible tips for strong aspiration and efficient clot removal. However, they lack flow arrest and do not feature a balloon to improve the system's stability.

The iVascular (Sant Vicenç dels Horts, Barcelona) neurothrombectomy devices, including the iNedit balloon distal access catheter, iNdeep microcatheter, and iNtercept stent retriever, are designed to leverage the benefits of temporary proximal blood flow restriction via a balloon located 5 cm from the catheter tip, providing enhanced stability during distal aspiration. These devices are compatible with all commonly used stent retrievers.

Preliminary results on using the iNedit balloon DAC in different clinical scenarios indicate that the device achieved a high first-pass effect and final recanalization rate with no safety concerns, resulting in a high percentage of favorable clinical outcomes (11).

This study aims to evaluate the efficacy and safety of this novel MT approach (SEMTiC strategy), which uniquely combines distal access, flow arrest, and flow reversal in a single procedure for acute ischemic stroke (AIS) patients. The non-inferiority of this combination will be compared to existing literature data.

## Methods and analysis

### Design

SEMTiC-01 is a prospective, multicenter, single-arm, open-label clinical safety and efficacy investigation.

### Study population

In the SEMTiC-01 trial, eligible participants are AIS patients treated within 24 h of symptom onset, defined as the last time the patient was seen well (LTSW; the start of the procedure is defined as arterial puncture time). Patients will be enrolled at 18 sites, which are in Spain (14 sites: Hospital Clinic Barcelona, Hospital Universitario de Bellvitge, Hospital Universitario Central de Asturias, Hospital Reina Sofía de Córdoba, Hospital Vall d'Hebron, Hospital Clínico San Carlos, Hospital German Trías i Pujol, Hospital Universitario de Cruces, Hospital General Universitario de Alicante, Hospital Universitario la Fe, Hospital Universitario General de Canarias, Hospital General Universitario Gregorio Marañón, Hospital Universitario de Badajoz, and Hospital Clínico Universitario de Valencia), Germany (3 sites: K. München Hospital, K. Nürnberg Hospital, and K. Ludwigsburg Hospital), and Belgium (1 site: AZ Groeninge Hospital) between July 2022 and June 2024.

The detailed inclusion/exclusion criteria are listed in Table 1.

**Table 1:** Study inclusion and exclusion criteria.

**Inclusion criteria**
**Clinical**
1. Age ≥ 18 years.
2. Informed consent signed by the patient or their representative to use the patient's data.
3. Focal disabling neurological deficit consistent with acute cerebral ischemia.
4. Baseline NIHSS obtained before the procedure of ≥6 points and ≤25 points.
5. Pre-stroke mRS score ≤2.
6. Planning to start treatment within 24 h of symptoms onset, defined as the LTSW (the start of the procedure is defined as arterial puncture).
**Neuroimaging criteria**
1. Occlusion (TICI 0 or TICI 1) of the internal intracranial carotid artery, M1 and M2 segments of the middle cerebral artery, and T carotid artery, suitable for mechanical thrombectomy confirmed by conventional angiography.
2. For patients treated ≤8 h:
a) Score 6–10 on Alberta Stroke Program Early CT Score (ASPECTS).
For patients treated between 8 and 24 h:
a) “Target Mismatch Profile” on CT perfusion or MRI (ischemic core volume was <70 mL, mismatch ratio was >1.8, and mismatch volume was >15 mL).
3. Ability to obtain selective angiography by catheterization of the target artery.
**Exclusion criteria**
**Clinical**
Initially treated with a different thrombectomy device in the same procedure.
1. Patient had suffered a stroke in the past year.
2. Occlusion (TICI 0 or TICI 1) in vertebrobasilar territory.
3. Clinical symptoms suggestive of bilateral stroke or stroke in multiple territories.
4. Known hemorrhagic diathesis, coagulation factor deficiency, or oral anti-vitamin K anticoagulant therapy with an INR > 3.0.
5. Blood glucose <50 or >400 mg/dL. Note: If blood glucose could be successfully reduced and maintained at an acceptable level by administering the medication recommended by the European Stroke Organization (ESO) in its guidelines, the patient might be included.
6. Severe sustained hypertension (systolic pressure >185 mm Hg or diastolic pressure >110 mm Hg). Note: If blood pressure could be successfully reduced and maintained at an acceptable level by administering the medication recommended by the ESO in its guidelines (also IV infusion of antihypertensives), the patient might be included.
7. Serious advanced or terminal illness with an anticipated life expectancy of <6 months.
8. History of life-threatening allergy (more than rash) to contrast medium.
9. Known allergy to nickel, prior to treatment.
10. Known renal insufficiency with creatinine ≥3 mg/dL or glomerular filtration rate (GFR) <30 mL/min.
11. Cerebral vasculitis.
12. Known current cocaine use.
13. Patient who was participating, at the time of inclusion, in a study with a device or drug that could affect this study.
14. Patients who were unlikely to be available for 90-day follow-up (e.g., no fixed home address and visitors from overseas).
**Neuroimaging criteria**
1. CT or MRI evidence of hemorrhage (the presence of microbleeds was allowed).
2. Significant mass effect with midline shift.
3. Evidence of complete occlusion, high-grade stenosis, or arterial dissection in the extracranial or petrous segment of the internal carotid artery (tandem lesions).
4. Patients with known or suspected underlying intracranial atherosclerotic lesions responsible for the target occlusion.
5. Patients with occlusions in multiple vascular territories (e.g., bilateral anterior circulation or anterior/posterior circulation).
6. Evidence of intracranial tumor except for asymptomatic meningiomas with no mass effect.

### Treatment devices

#### SEMTiC strategy

This approach leverages an innovative balloon distal design, where a balloon located a few centimeters from the catheter tip blocks blood flow, while the distal tip enables aspiration. This technique uniquely integrates distal access, flow reversal, and flow arrest—via the distal balloon—into a single procedure.

##### iNedit

The iNedit balloon distal access catheter is a double-lumen coaxial catheter from the connector to the balloon and a single lumen from the balloon to the tip. It is designed with braid and coil reinforcement. The distal segment has a compliant occlusion balloon located 5 cm from the tip of the catheter, the purpose of which is to occlude the flow at the discretion of the operator, if deemed necessary during the procedure.

The catheter's proximal “Y”-shaped luer-lock connector has a side port for balloon inflation/deflation with diluted contrast and a straight port for device passage or aspiration. The outer diameter of the catheter is compatible with access systems with a minimum internal lumen of 0.088” (2.235 mm). The maximum volume of balloon inflation is 0.2 mL.

##### iNdeep

The iNdeep microcatheter is a single-lumen catheter from proximal to distal shaft, reinforced with braid and variable stiffness through its entire length to ensure optimal trackability. At the most distal end, there is a radiopaque marker that helps to visualize the device under fluoroscopy.

The catheter's distal body has a durable hydrophilic coating for easier navigation through tortuous arteries and is available in three internal diameters (0.017”, 0.021”, and 0.027”).

##### iNtercept

The stent retriever consists of a self-expanding nitinol basket with proximal and distal markers for fluoroscopic positioning and gold markers to assess deployment. It is supplied compressed in an introducer sheath for easy insertion via a microcatheter. After full deployment, the device is deployed within or beyond the thrombus and retracted under suction.

### Standard protocol approvals, registration, and patient consent

The SEMTiC-01 trial is registered on clinicaltrials.gov (NCT05893719). The protocol and data collection of the trial have been approved by the Hospital Clinic, Barcelona, the ethics committee, and all participating sites. Written informed consent will be obtained from patients or representatives before inclusion into the trial.

### Patients inclusion

All patients were consecutively included when the interventional neurologist confirmed that they met all the inclusion criteria and would treat them with the proposed strategy (SEMTiC strategy), including the use of all three devices. In cases where successful reperfusion (defined as eTICI 2b-3) was not achieved after three passes with the iNdeep, iNedit, and iNtercept devices [all with European Conformity (CE) market], rescue therapy—defined as the use of any additional strategies or devices—was permitted.

Deferred informed consent was utilized to prevent delays in treatment initiation. Once a patient is considered eligible for the study, the patient or their representative receives the “Patient Information Sheet” and “Informed Consent Form” (ICF). Written consent will be required from the patient or a family member during the study period to authorize the use of patient data. Investigators will explain the study and deliver a copy of the signed ICF. Each consented patient will be assigned a unique identifier, composed of a site number and a sequential patient number, based on the order of inclusion at each site.

### Completion of the study

All patients treated (with the study device or rescue therapy) were evaluated at 24, 72 h, or discharge and 90 days after the index procedure. The patient's study participation is considered complete at the 90-day visit.

### Safety outcome

The Clinical Events Committee (CEC) reviews all adverse events to determine their severity and seriousness (SAE) and to adjudicate causality related to the procedure or the device. The latter is assessed using the following scale: “possible” (the relationship with the use of the investigational device or the relationship with procedures, is weak but cannot be ruled out completely), “probable” (the relationship with the use of the investigational device or the relationship with procedures, seems relevant and/or the event cannot be reasonably explained by another cause), and “causal relationship” (the serious adverse event is associated with the investigational device or with procedures beyond reasonable doubt).

### Primary outcomes

#### The primary efficacy endpoint

The primary efficacy endpoint is the achievement of a successful neurothrombectomy, defined as a revascularization of grade ≥2b-3 with ≤3 passes on the eTICI scale (12). The Core Lab used the eTICI scale to assess the performance of the overall system.

#### The primary safety endpoint

For the primary safety endpoint, all serious adverse events (SAEs) within 24 h (−8/+12 h) and mortality at 90 days were considered.

### Secondary outcomes

The secondary efficacy endpoint variable includes (I) good functional outcome (mRS 0–2 at 90 days post-treatment); (II) rapid neurological improvement [National Institutes of Health Stroke Scale (NIHSS) reduction >4 points or NIHSS ≤4 within 24 h]; (III) improvement at 72 h (NIHSS reduction ≥8 points or NIHSS 0–1 at 72 h or at discharge); (IV) procedure duration (time from a puncture to achieving eTICI grade ≥2b in fewer than 3 passes or final angiogram); (V) the number of passes required for recanalization; percentage of effective recanalization on the first pass; and (VI) navigability (i.e., the microcatheter and the distal access balloon catheter to reach the occlusion in the main vessel to allow navigation and deployment of the stent retriever to carry out the neurothrombectomy), assessed on a scale as “good”, “standard”, and “deficient”.

Secondary safety endpoint variable includes (I) intracerebral hemorrhage (ICH) assessed by magnetic resonance imaging (MRI)/computed tomography (CT) at 24 h, with symptomatic intracerebral hemorrhage (sICH) defined as ICH causing clinical deterioration (NIHSS worsening by ≥4 points) or death; (II) neurological deterioration (≥4 point increase on the NIHSS scale at 24 h); (III) distal embolization, i.e., any anterograde occlusion in the distal to the target artery injury, in the target ischemic territory; (IV) occurrence of embolization in previously unaffected areas on cerebral angiography; (V) mortality rate (death related to the procedure within 3 days or at discharge); (VI) procedure complications (arterial perforation and dissection—specifically involving the internal carotid artery (ICA), vasospasm, and embolization in new vascular territories); and (VII) infarction rate in previously unaffected areas (evaluated by MRI/CT 24 h post-procedure).

### Follow-up procedure

Study evaluations will be conducted at three time points: 24 h, 3 days (or at discharge), and 90 days following MT.

At 24 h post-MT (−8/+12 h), the following assessments will be performed, and relevant data will be collected: NIHSS, control neuroimaging (CT or MRI), recording of any adverse events (AEs) or SAEs, and documentation of concomitant medications.

At 3 days post-MT (±24 h) or at discharge, whichever occurs first, the following assessments will be performed: NIHSS, recording of any relevant AEs or SAEs, and determination of stroke etiology.

At 90 days post-MT, assessments will preferably be conducted through a face-to-face visit, although a telephone visit is also acceptable. Data collected will include the mRS and any SAEs resulting in death.

### Sample size determination

We anticipated that 82% of patients would achieve eTICI ≥ 2b, compared to 71.1% reported in previous research, to estimate the sample size in a non-inferiority study (13). To ensure 98% statistical power in a *Z*-test with a one-sided significance level of 0.025 and a non-inferiority margin of −2.6%, an interim analysis with 115 patients is needed (providing 51.1% cumulative information), assuming a 15% withdrawal rate. However, the sample size was increased to *N* = 225 to gather additional real-world clinical data.

### Statistical analysis

All data will be analyzed with SPSS 27.0 Software. The mean ± standard deviation (SD) will be used if the data are normally distributed, and the median and interquartile range (IQR) will be used if the data are non-normally distributed.

The proportion of patients with eTICI ≥ 2b will be obtained, and the significance level for non-inferiority will be determined using the *Z* statistic for comparing two proportions with a non-inferiority margin of −0.026.

All values will be estimated using the 95% confidence interval (recommended Wilson's or Agresti methods). The following study populations will be defined for statistical analysis:

- Intention to treat (ITT) is defined as all patients included in the study who have been treated with at least one of the study devices. The primary efficacy analysis in relation to non-inferiority will be performed on this population.
- Population by protocol (PP) is defined as ITT subset with no major deviations from the clinical investigational plan. The primary efficacy analysis in relation to non-inferiority will also be performed with this population.
- Modified population by protocol (PP) is defined as PP subset excluding patients treated with rescue therapy.

All outcomes will be evaluated across all study populations. The proportion of patients undergoing rescue therapy will also be measured. The impact of rescue therapy on both angiographic and clinical outcomes will be assessed using adjusted logistic regression models. A subgroup analysis will compare the results achieved in cases with the occlusion of the M2 vs. the M1/T carotid segments.

### Trial status

The analysis after 115 patients showed non-inferiority of the SEMTiC strategy using iVascular devices in achieving the primary efficacy endpoint (80% in ITT and 89% in PP populations). These 115 patients had enough statistical power to finish recruitment at that point. However, the increase in the sample size to *N* = 225 was designed to obtain additional real-life clinical data. The study completed recruitment on June 30, 2024.

## Discussion

The primary efficacy endpoint of this study is to evaluate the non-inferiority of the iNedit, iNdeep, and iNtercept thrombectomy system in treating acute ischemic stroke in terms of successful recanalization, compared to existing literature. The key efficacy measure (eTICI ≥2b) is a well-established predictor of functional outcomes after mechanical thrombectomy (8). Literature reports successful recanalization rates ranging from 59 to 88% (14, 15).

For the primary safety outcome, we will analyze 90-day mortality, which has been reported to range from 7.9 to 14% in prior studies (3, 9, 15–17), along with any possible serious adverse event as evaluated by an external safety committee.

The secondary prespecified outcome will include eTICI ≥2c following MT, considering that the current goal is to achieve the highest possible percentage of complete or near-complete recanalization (2c or 3). ARISE II trial reported a rate of 64.8% for eTICI ≥2c (3). Favorable clinical outcomes, as measured by mRS of 0–2 at 90 days, will still be evaluated. In previous trials, ~50% of patients achieved this outcome 90 days post-MT (3, 9, 10, 16, 18).

In analyzing secondary outcomes (secondary efficacy outcome V), particular emphasis will be placed on evaluating the TICI score and the degree of recanalization achieved at each pass with the three devices. In addition, a comparative assessment of outcomes with and without rescue therapy will be conducted to understand its impact on procedural success.

Moreover, given the innovative nature of the devices (secondary efficacy outcome VI), thorough attention will be dedicated to evaluating procedure-related complications, including arterial perforation, dissection (particularly involving the ICA), vasospasm, and embolization in new vascular territories. In particular, the risk of balloon rupture will be considered a specific complication associated with using iNedit catheters. It will be recorded and reported during the trial as a *post-hoc* analysis, even if it is expected to have no clinical relevance.

These complications will be systematically assessed by the local investigators, independently reviewed through imaging analysis by the external core lab, and adjudicated by an independent Clinical Events Committee (CEC) to ensure objective evaluation and accuracy.

Special attention will be given to outcomes related to the navigability of the devices as the balloon catheter has been specifically developed to improve the navigability compared to currently available balloon guide catheters. Previous studies of preliminary experiences with this device have reported high scores in terms of navigability (11).

In addition, we expect a lower incidence of vasospasm, as balloon inflation occurs at the petrous segment of the carotid artery, where the periosteal layer provides stability by attaching to both the vessel and the surrounding bone. This contrasts with the cervical ICA, which is more prone to collapse due to being surrounded by the neck's soft tissues (19).

Integrating distal access, flow reversal, and flow arrest in mechanical thrombectomy potentially enhances clot retrieval efficiency, reduces embolization risk, and improves procedural safety. DACs allow closer proximity to the thrombus, providing stable support for thrombectomy devices, improving clot retrieval, and facilitating direct aspiration. Flow reversal helps prevent clot fragmentation and distal migration during retrieval, while flow arrest enables safer retrieval of large thrombi and enhances overall procedural control.

A potential limitation of the strategy proposed by the current study is that the distal aspiration balloon catheter has an inner diameter of 0.058”, which refers to the width of the hollow tube that runs through the center of the device. This may not be considered large enough to perform aspiration in certain cases with large clots, such as in the carotid T, according to some operators. However, this inner dimension may be advantageous for aspiration in distal vessels (20). A subgroup analysis comparing outcomes in M2 occlusions vs. the carotid T and M1 segments could support the future use of this catheter specifically for M2 occlusions, especially by inflating the distal balloon to achieve more effective flow arrest.

The main objective of this study is to demonstrate the feasibility and safety of the combined use of the three iVascular devices. The results of this study should be interpreted with caution due to its single-arm design, which may limit the ability to determine whether an alternative method would have yielded superior outcomes. However, a multicenter international study design has been implemented to reduce center-specific biases and enhance the generalizability of the findings. Furthermore, a secondary propensity score matching analysis will account for potential confounding factors by comparing similar patients treated with alternative methods.

The results of the SEMTiC studies, expected to be available in 2025, will answer whether combining flow arrest, flow reversal, and distal access (SEMTiC strategy) is an effective and safe strategy for performing mechanical thrombectomy.
